# An Electrochemical Sensor for the Determination of Trace Concentrations of Cadmium, Based on Spherical Glassy Carbon and Nanotubes

**DOI:** 10.3390/ma16083252

**Published:** 2023-04-20

**Authors:** Malgorzata Grabarczyk, Cecylia Wardak, Robert Piech, Agnieszka Wawruch

**Affiliations:** 1 Department of Analytical Chemistry, Institute of Chemical Sciences, Faculty of Chemistry, Maria Curie-Sklodowska University, 20-031 Lublin, Poland; cecylia.wardak@mail.umcs.pl (C.W.);; 2 Faculty of Materials Science and Ceramics, AGH University of Science and Technology, Mickiewicza 30, 30-059 Krakow, Poland; rpiech@agh.edu.pl

**Keywords:** spherical glassy carbon microparticle powder, multiwall carbon nanotubes, electrochemical sensor, bismuth film electrode, stripping voltammetry, cadmium

## Abstract

The practical application of a novel, eco-friendly electrochemical sensor based on low-dimensional structures, spherical glassy carbon microparticles, and multiwall carbon nanotubes is described. This sensor, modified with a bismuth film, was used for the determination of Cd(II) by the anodic stripping voltammetric method. The instrumental and chemical factors influencing the sensitivity of the procedure were thoroughly investigated and their most favorable values were selected (acetate buffer solution pH = 3 ± 0.1; 0.15 mmol L^−1^ Bi(III); activation potential/time: −2 V/3 s; accumulation potential/time: −0.9 V/50 s). Under the selected conditions, the method exhibited linearity in the range of 2 × 10^−9^ to 2 × 10^−7^ mol L^−1^ Cd(II) with a detection limit of 6.2 × 10^−10^ mol L^−1^ Cd(II). The results obtained also showed that the application of the sensor for Cd(II) detection did not experience any significant interference in the presence of a number of foreign ions. The applicability of this procedure was evaluated using TM-25.5 Environmental Matrix Reference Material and SPS-WW1 Waste Water Certified Reference Material as well as river water samples through addition and recovery tests.

## 1. Introduction

Cadmium does not occur in the natural environment in large quantities; we find it in zinc, copper, lead, or sulfide ores. Their extraction and processing release significant amounts of cadmium into the atmosphere, water, and soil. In industry, cadmium is used in the manufacture of dyes and stabilizers for plastics as well as in galvanic protective coatings, solders, alloys, and cadmium rods. It is a component of nickel–cadmium batteries, fireworks, and fluorescent paints. Another major source of cadmium release to the environment is the combustion of fossil fuels (including hard coal), which results in the formation of large amounts of cadmium oxide. This oxide is easily soluble in water and, therefore, it enters the ecosystem through aquatic organisms and plants. A significant source of cadmium in the environment is artificial fertilizers (e.g., superphosphates), which are contaminated with this metal in amounts ranging from 10 to 100 mg/kg. Their long-term and widespread use leads to cadmium contamination of the soil. This is why monitoring the cadmium content in various environmental samples is such a key issue. Specifically, cadmium is among the elements that exhibit particularly toxic effects on the human body and other living organisms, as well as plants. It causes the most damage in organs where it easily accumulates, i.e., the liver, kidneys, and bones. Acute poisoning caused by a single high dose of the metal in humans is rare; poisoning caused by long-term exposure to even small concentrations of cadmium is far more common [1,2,3,4,5,6,7,8]. Unsurprisingly, there are numerous papers in the literature devoted to the determination of low concentrations of cadmium in a variety of matrices. Voltammetric methods, which are characterized by relatively low instrument cost but also by high sensitivity, are one of the more commonly used techniques. The development of these methods is currently focused on finding new working electrodes and improving existing ones.

The first information about glassy carbon (GC) appeared in the mid-1950s, and in 1965, a new form of carbon known as “glassy” carbon was used as an indicator electrode in voltammetry [9]. Glassy carbon is a gas-impermeable, electrically conductive material that is highly resistant to chemical attack. As described, it is suitable for use over the potential range from about +1.2 to −0.8 V in acid medium, and for most tested ions, a peak-type wave that approaches ideality is obtained [9,10]. The importance of glassy carbon in voltammetry is confirmed by the fact that to this day it is a commonly used electrode material [11,12,13,14]. One of the recently described applications of GC is its use as a perfect substrate to create metallic film electrodes, e.g., bismuth film electrode (BiFE). Bismuth film electrodes are currently a very good solution for the determination of trace amounts of analytes, especially as a replacement for toxic mercury electrodes. BiFEs exhibit several attractive properties such as low toxicity, simple preparation, and high sensitivity, and they generate well-defined electrochemical signals [15,16,17,18,19,20]. This is also the case in the trace analysis of cadmium, as evidenced by the number of articles devoted to using BiFEs as working electrodes. In all of these works, the BiFE was generated on glassy carbon [21,22,23,24,25,26,27,28,29,30,31,32]. The aim of our research was to develop a new electrode that uses the advantages of glassy carbon and currently commonly available carbon nanomaterials, and to use it as a substrate to create a bismuth film electrode in order to increase the sensitivity of cadmium determinations.

Our research was focused on the search for materials with low-dimensional structures, known for their large surface area and small spatial dimensions, enabling the development of an ideal substrate for generating a bismuth film. For this purpose, a mixture of spherical glassy carbon (SGC) microparticle powder and multiwall carbon nanotubes (CNTs) was used, which turned out to be an ideal substrate for generating the bismuth film and, through its developed surface area, allowed lowering the detection limit of cadmium compared to classical glassy carbon serving as a substrate. Spherical glassy carbon microparticles come in many sizes and microspheres are generally defined as spherical particles greater than 1 micron and smaller than 1000 microns in diameter. As proven in [33], which examined the influence of glassy carbon microparticles of various sizes on the properties of electrodes, the limits of detection decrease with decreasing size of glassy carbon spherical microparticles, with the best results achieved for glassy carbon spherical microparticles with a diameter of 0.4–12 µm. This was also confirmed by other voltammetric procedures that used spherical microparticles with a diameter of 0.4–12 µm in the electrodes [34,35,36]. Therefore, this size of carbon microparticles was also used in our study. Nanotubes, which represent a 1D system, i.e., two dimensions are given in the nanoscale, are another material based on low-dimensional systems that was used as a component of our electrodes. A carbon nanotube is a rolled-up graphene plane. The properties of carbon nanotubes depend on the way they are rolled up, their length, diameter, and morphology, as well as the defects present. There are two basic types of nanotubes: single-walled with a diameter of 0.8–2 nm and multi-walled usually with a diameter of 5–20 nm, which are made of concentrically arranged single-walled nanotubes. The application of nanotubes as an electrode material began with research published in 1996 by Britto [37]. Since then, there has been rapid development in the use of nanotubes in electrochemical sensors [38,39,40]. CNTs have unique properties such as large specific surface area, high charge transfer capacity, hydrophobicity, and chemical stability. They facilitate electron transfer and enhance the signal/noise ratio, resulting in improved analytical parameters of CNT-modified electrodes, which directly translates into an increase in the sensitivity of determinations [41,42,43].

These unique properties of spherical glassy carbon microparticles and multiwall carbon nanotubes were used in the electrochemical sensor proposed in this work, which was proven to be an ideal material for creating a bismuth film electrode for use in the voltammetric determination of trace amounts of cadmium in environmental waters. It was evidenced by numerous papers devoted to the issue of determining trace amounts of cadmium by voltammetric methods. The first studies were based mainly on mercury electrodes, but with increasing awareness of their toxicity, other electrode materials were used. In recent years, bismuth film electrodes, which account for a significant share of cadmium voltammetry research, have been increasingly mentioned [21,22,23,24,25,26,27,28,29,30,31,32]. Therefore, our work was focused on further improving the procedure for the voltammetric determination of cadmium on a bismuth firm electrode by using a novel substrate for its generation.

## 2. Materials and Experimental Work

### 2.1. Chemicals

Analytical grade reagents were employed without further purification. Water used for all purposes was distilled from a Milli-Q water purification system (Millipore, UK). Standard solutions of 1 g L^−1^ Bi(III) and Cd(II) were acquired from Fluka (Buchs, Switzerland). A standard cadmium(II) solution was prepared every day to the desired concentrations of 1 × 10^−5^ and/or 1 × 10^−6^ mol L^−1^ by dilution with distilled water. TM-25.5 Environmental Matrix Reference Material and SPS-WW1 Waste Water were acquired from Environment and Climate Change Canada and Spectrapure Standards AS (Oslo, Norway), respectively. Multiwall carbon nanotubes with O.D. × I.D. × L of 10 nm ± 1 nm × 4.5 nm ± 0.5 nm × 3~6 μm were obtained from Sigma-Aldrich (St. Louis, MO, USA). Spherical glassy carbon powder size 0.4–12 µm was obtained from HTW Hochtemperatur-Werkstoffe GmbH (Thierhaupten, Germany).

### 2.2. Apparatus

Stripping voltammetry was carried out on a µAutolab (Eco Chemie, Utrecht, The Netherlands) with a personal computer operated by GPES 4.9 software. All measurements were carried out using a 10 mL quartz cell. The three-electrode array consisted of an Ag/AgCl electrode filled with saturated NaCl as a reference electrode, a platinum counter electrode, and a multiwall carbon nanotubes/spherical glassy carbon (CNTs/SGC) electrode as a working electrode. The morphology of the CNTs/SGC electrode was characterized using a Nikon ECLIPSE MA200 inverted metallurgical microscope (Tokyo, Japan) and a Quanta 3D FEG high-resolution scanning electron microscope (FEI Hillsboro, Hillsboro, OR, USA).

### 2.3. CNTs/SGC Electrode Fabrication

**Figure 1:**
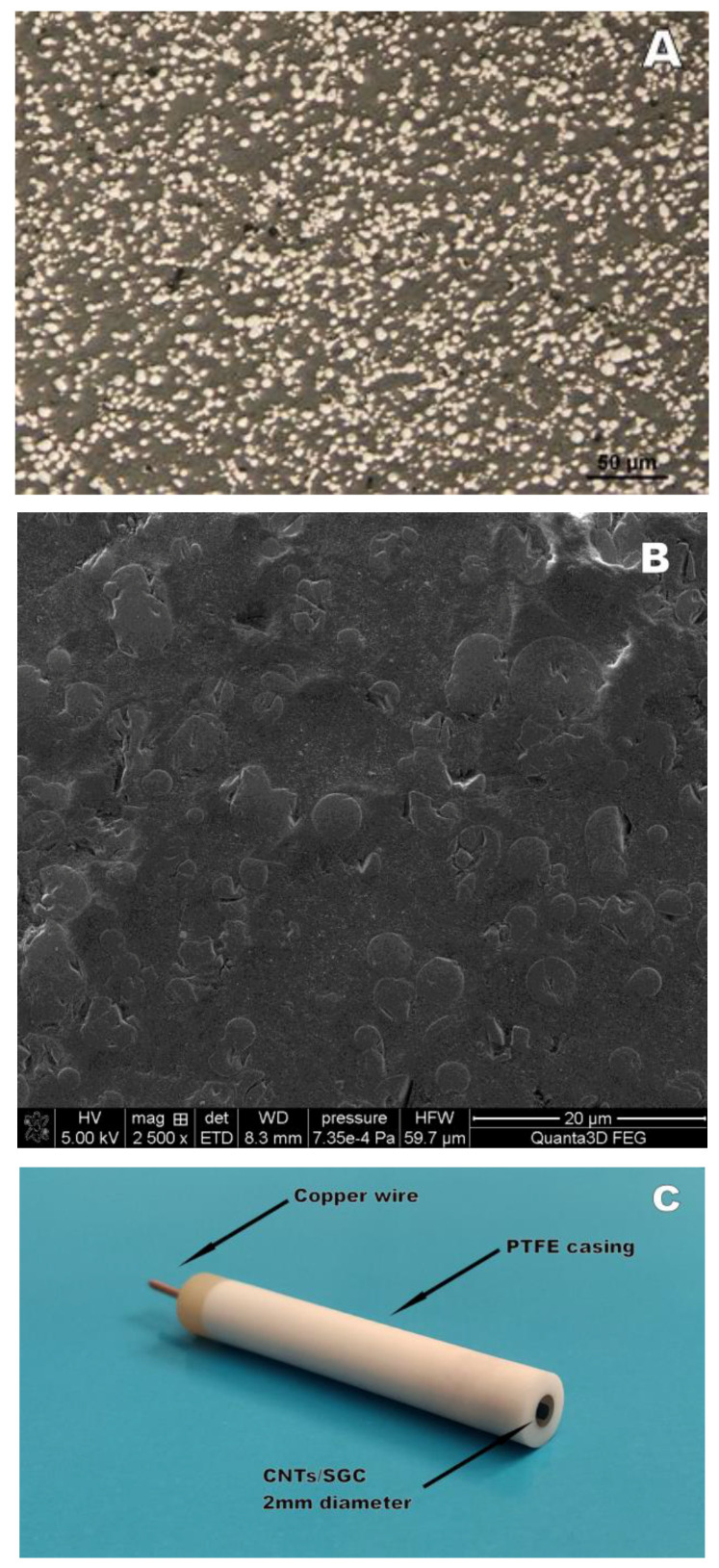
A microscopic image of the CNTs/SGC electrode in 50 µm scale obtained by the Nikon ECLIPSE MA200 inverted metallurgical microscope (**A**). A microscopic image of the CNTs/SGC electrode in 20 µm scale obtained by the Quanta 3D FEG high-resolution scanning electron microscope (**B**). A picture representing the construction of the electrode (**C**).

The CNTs/SGC electrode was prepared in two steps, first by mixing the multiwall carbon nanotubes with epoxy resin (in a ratio of 1:25) to a homogeneous mass. The resulting mass was heated to 115 degrees Celsius and hot-centrifuged to remove air bubbles. Then, in the second stage, this mass containing multiwall carbon nanotubes was mixed with spherical glassy carbon powder (size 0.4–12 µm) in a 2:1 ratio. The mixture of multiwall carbon nanotubes and spherical glassy carbon powder obtained in this way was placed under pressure in a housing made of PEEK in a hole with a diameter of 2 mm. Copper wire was used to provide electrical contact. Finally, the CNTs/SGC electrode was polished first with coarse (P120) and then fine (P2000) sandpapers, rinsed with plenty of water, and placed in an ultrasonic bath (Sonic-3, Polsonic, Poland) for 30 s to remove polishing material residues. On each day before the measurements, the electrode was polished with a 0.3 µm suspension of aluminum oxide on a Buehler polishing pad for 30 s and then immersed in an ultrasonic bath for 30 s to remove aluminum oxide. The morphology of the CNTs/SGC electrode was investigated using the inverted metallurgical microscope. A microscopic image of the CNTs/SGC electrode at the µm scale is shown in Figure 1. The bright elements of irregular shape and size correspond to the spherical glassy carbon microparticles with 0.4–12 µm particle size and the multiwall carbon nanotubes mixed with epoxy resin are represented by the darker surface.

### 2.4. Certified Reference Materials and River Water Sample Preparation

TM-25.5 Environmental Matrix Reference Material and SPS-WW1 Waste Water were analyzed without any preparation after appropriate dilution. Taking into account the fact that both materials were preserved with HNO_3_ (TM-25.5—0.2% HNO_3_ and SPS-WW1—0.5% HNO_3_) to neutralize the pH, an appropriate amount of NaOH was additionally introduced into the analyzed solution.

Water samples from the Bystrzyca and Bychawka Rivers were collected and stored in polypropylene bottles at a temperature of 6 °C. The collected samples were submitted to analysis after minor pretreatment consisting of filtration through a 0.45 μm cellulose acetate membrane filter. Recovery tests were carried out using river water samples spiked with standard solutions of the required concentrations of Cd(II) about two hours before filtering.

### 2.5. Electrochemical Analysis Procedure

Unless otherwise stated, 0.1 mol L^−1^ acetate buffer solution with pH = 3 ± 0.1 was adopted as the supporting electrolyte. To produce the bismuth film, 1.5 × 10^−4^ mol L^−1^ Bi(III) was introduced into the supporting electrolyte. The quantitative determination of Cd(II) concentration in the solution was performed with differential pulse voltammetry (DPV) from −1.0 to −0.4 V using the following parameters: a potential scan rate of 40 mV s^−1^ and a pulse height of 50 mV. An accumulation potential of −0.9 V with an accumulation time of 50 s was preceded by an activation potential of −2 V for 3 s. In the accumulation stage, the formation of the bismuth film and the accumulation of cadmium as a result of its reduction to the metallic form occurred simultaneously while the signal obtained by changing the electrode potential in the positive direction was the result of the oxidation of Cd(0) to Cd(II). Applying the activation potential before the actual accumulation step allowed obtaining higher cadmium peaks in the voltammogram. After each measurement, an electrochemical cleaning process was applied to the electrode at 0.2 V for 20 s under stirring conditions to remove residual metals. All measurements were executed at ambient temperature in the presence of oxygen.

### 2.6. Stability, Repeatability and Reproducibility

The stability, repeatability, and reproducibility of the electrode response are important parameters from the point of view of analytical application. The long-term stability of the CNTs/SGC electrode was studied over three months. The measurements with the electrode were performed in the 5 × 10^−8^ mol L^−1^ Cd(II) standard solution, 0.1 mol L^−1^ acetate buffer, and 1.5 × 10^−4^ mol L^−1^ Bi(III). The voltammograms were registered every week and the relative standard deviation (RSD) was calculated based on the values of the peak currents in the recorded voltammograms, which was found to be 5.1%. The repeatability was assessed by recording six measurements using the CNTs/SGC electrode, one after the other, and calculating the relative standard deviation based on the recorded cadmium peak currents. The RSD was 3.1%. The reproducibility was investigated by determining 5 × 10^−8^ mol L^−1^ Cd(II) using three independently fabricated CNTs/SGC electrodes. Four measurements were made for each electrode and the calculated relative standard deviation was 5.5%.

## 3. Results and Discussion

### 3.1. Characteristics of CNTs/SGC Electrode

In order to select the optimal CNTs:SGC ratio, a homogeneous mass was prepared by mixing the multiwall carbon nanotubes with epoxy resin in a ratio of 1:25. This mass was then mixed with spherical glassy carbon microparticles in the following proportions: 1:1, 1:2, 2:1, and 3:1. In this way, four types of electrodes differing in the ratio of CNTs to SGCs were obtained. Then, three voltammograms were recorded for each of the electrodes in a standard solution containing 5 × 10^−8^ mol L^−1^ Cd(II) using the standard procedure described in Section 2.5 Electrochemical analysis procedure. The highest averaged cadmium peak current was obtained for the 2:1 ratio, so this electrode composition was chosen for further study.

The morphology of the CNTs/SGC electrode was investigated using the inverted metallurgical microscope and the high-resolution scanning electron microscope. A microscopic image of the CNTs/SGC electrode at the 50 µm scale is shown in Figure 1A. The bright elements correspond to the spherical glassy carbon microparticles with 0.4–12 µm particle size, as guaranteed by the producer, and the darker surface corresponds to the multiwall carbon nanotubes mixed with epoxy resin. A scanning electron microscopic image of the CNTs/SGC electrode at the 20 µm scale is shown in Figure 1B. The spherical shape of the glassy carbon is also clearly visible in the SEM images, and it is clear that most of them are around 1 µm in diameter. Unfortunately, it was not possible to clearly observe the nanotubes in both images, but this is understandable given that their size in two dimensions is on the order of a few nm. A picture representing the construction of the electrode is presented in Figure 1C.

### 3.2. Studies of the Optimal Solution Composition

**Figure 2:**
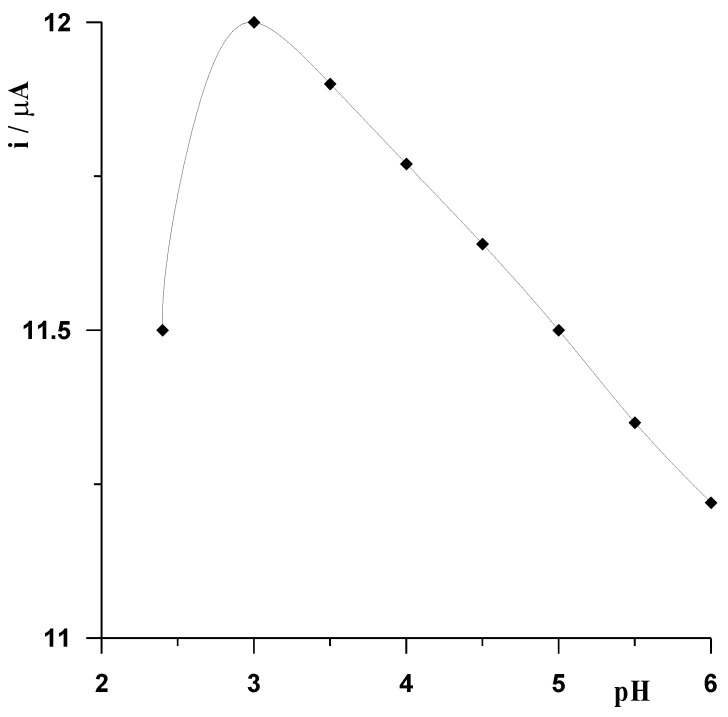
The influence of pH on the voltammetric signal of 5 × 10^−8^ mol L^−1^ Cd(II). In the case of pH = 2.4, acetic acid was used, in the case of other pHs, acetate buffer was used.

The first step in the selection of the optimal conditions for the determination of Cd(II) using the CNTs/SGC electrode modified with a bismuth film was to select the composition of the solution from which the measurement was carried out. The first necessary component was the supporting electrolyte ensuring the conductivity of the solution, the elimination of the migration current, and the appropriate pH. When analyzing ASV procedures for the determination of Cd(II) based on BiFEs described in the literature, acetic acid or acetate buffer with a pH up to 5 was most often used as the supporting electrolyte [21,22,24,25,26,28,29,30,31]. In our case (Figure 2), we found that the acetate buffer with pH = 3 was the best choice for the primary electrolyte. However, it should be noted that within the studied pH range, the signal changed only slightly. We used acetate buffer with pH = 3 ± 0.1 in all measurements.

**Figure 3:**
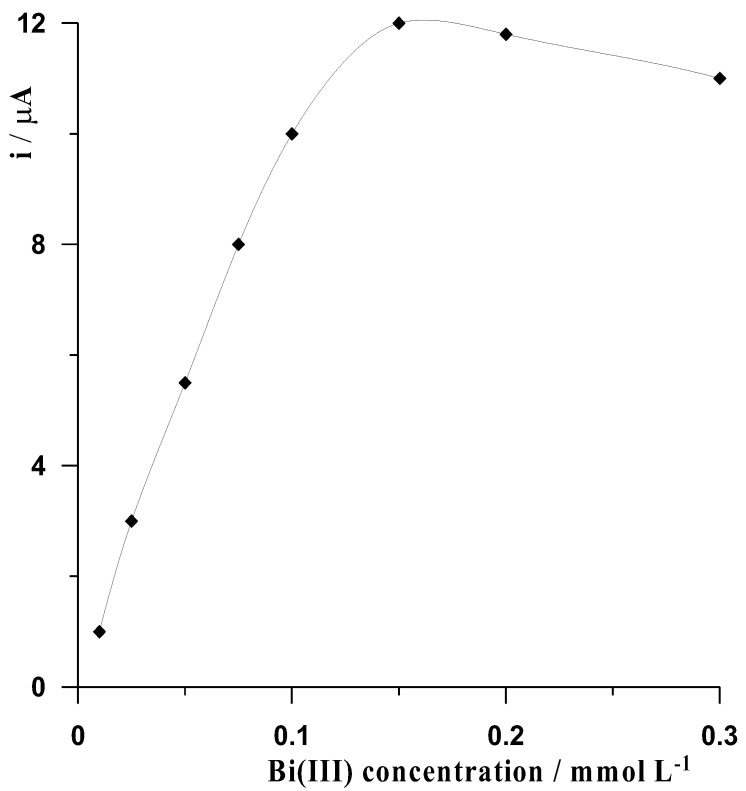
The influence of Bi(III) concentration on the voltammetric signal of 5 × 10^−8^ mol L^−1^ Cd(II). Supporting electrolyte is 0.1 mol L^–1^ acetate buffer at pH = 3 ± 0.1.

Bi(III) ions, from which a film of bismuth was formed during the measurement as a result of its reduction to metallic form, were another component of the solution in measurements based on in-situ formed BiFEs. Therefore, the behavior of the cadmium signal as a function of Bi(III) concentration was investigated. It was observed that with increasing concentration of bismuth ions in the solution, the cadmium peak gradually increased until a concentration of 0.15 mmol L^−1^, at which point it reached a maximum value. This concentration was taken as the optimal value because the cadmium peak began to gradually decrease for Bi(III) concentrations above 2 mmol L^−1^. Measurements made to select the optimal concentration of bismuth were carried out in a solution of 0.1 mol L^−1^ acetate buffer containing 5 × 10^−8^ mol L^−1^ Cd(II), and the results are shown in Figure 3.

### 3.3. Studies of Accumulation Potential and Time

**Figure 4:**
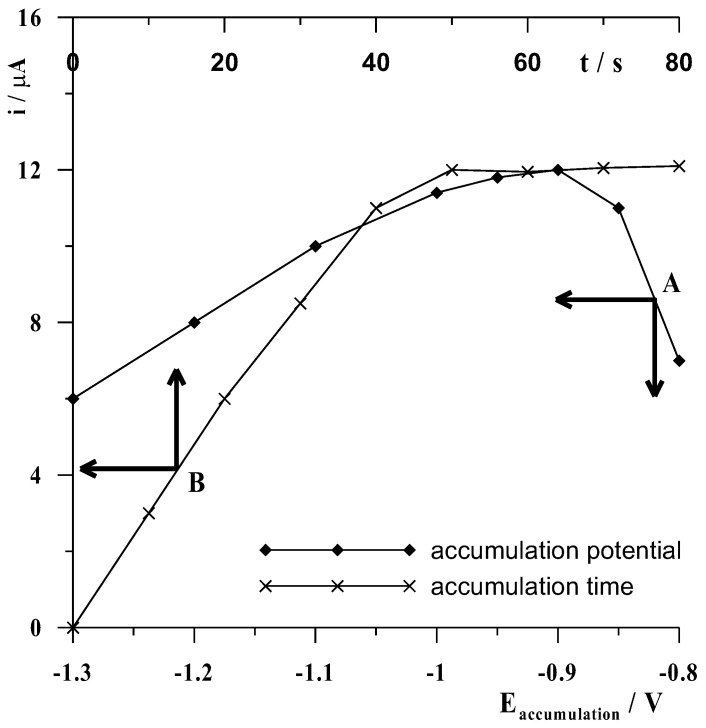
The influence of accumulation potential at a constant accumulation time of 50 s (**A**) and accumulation time at a constant accumulation potential of −0.9 V (**B**) on the voltammetric signal of 5 × 10^−8^ mol L^−1^ Cd(II). Concentrations of 0.1 mol L^–1^ acetate buffer at pH = 3 ± 0.1 and 1.5 × 10^−4^ mol L^−1^ Bi(III).

The accumulation potential and time in the proposed anodic voltammetric procedure play a dual role, affecting both the formation of the bismuth film and the cadmium metal accumulation efficiency, which are simultaneous processes. Thus, in order to select the optimal accumulation potential, a series of measurements was carried out at fixed concentrations of Cd(II) and Bi(III) ions in a solution of 0.1 mol L^−1^ acetate buffer, varying its value from −1.3 to −0.8 V for a constant time of 50 s. The results obtained are shown graphically in Figure 4 curve A as the dependence of the cadmium peak current on the value of the accumulation potential. As can be seen, the highest signals were obtained for an accumulation potential of −0.9 V, whereas the cadmium peak current gradually decreased at less and more negative potentials. Therefore, the potential of −0.9 V was chosen as the most suitable. For the same solution, another series of measurements was then carried out, this time at a constant accumulation potential of −0.9 V and varying its time from 0 to 80 s with a frequency of 10 s increments. The results obtained are shown graphically in Figure 4 curve B as the dependence of the cadmium peak current on the value of the accumulation time. A gradual increase in the cadmium signal was seen as the accumulation time increased to 50 s, but the cadmium peak current did not increase further above this time.

**Figure 5:**
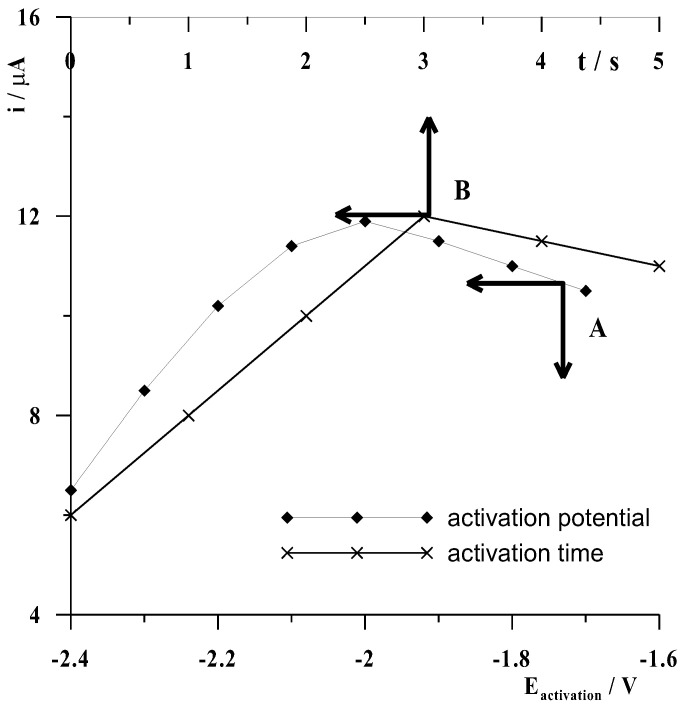
The influence of activation potential at a constant activation time of 3 s (**A**) and activation time at a constant activation potential of −2 V (**B**) on the voltammetric signal of 5 × 10^−8^ mol L^−1^ Cd(II). Concentrations of 0.1 mol L^−1^ acetate buffer at pH = 3 ± 0.1 and 1.5 × 10^−4^ mol L^−1^ Bi(III).

Based on previous experience [44,45], it was observed that a favorable effect on increasing the signal size of the analyte being determined could be achieved, when using metallic film electrodes created in situ, by applying a high negative potential to the electrode for several seconds before the accumulation step. Thus, it was investigated whether and to what extent application of a more negative potential (activation potential) to the CNTs/SGC electrode directly before the potential accumulation step would enhance the cobalt signal on the voltamperogram. The experiments were performed using the 5 × 10^−8^ mol L^−1^ Cd(II) solution, 0.1 mol L^–1^ acetate buffer, and 1.5 × 10^−4^ mol L^−1^ Bi(III), with an accumulation potential of −0.9 V and a constant time of 50 s preceded by an activation potential varying from −1.7 to −2.4 V with a constant time 3 s. The results obtained are presented in Figure 5 curve A showing the dependence of the cadmium peak current on the electrode activation potential. In analyzing these data, it was clear that the application of a −2 V activation potential had by far the most favorable effect on increasing the cadmium peak current. The next step was to select the duration of the activation potential and, therefore, experiments were carried out as described above, only this time the activation potential was fixed at 2 V and its duration was varied from 0 to 5 s with a frequency of 1 s increments. The results obtained are illustrated in Figure 5 curve B showing the dependence of the cadmium peak current on the duration of the activation potential at −2 V. As can be seen, the highest signal was obtained for an activation time of 3 s; therefore, all measurements were carried out using an activation step directly before the accumulation step as a result of applying a potential of −2 V to the electrode for 3 s.

### 3.4. Selectivity

The selectivity was evaluated based on the influence of different foreign ions on the electrochemical response of cadmium at the CNTs/SGC electrode modified with a bismuth film. The current responses of 5 × 10^−8^ mol L^−1^ Cd(II) were obtained before and after the addition of different foreign species into the solution under the optimal conditions. The interference threshold was set at above ± 5% change in peak cadmium current. No change in the cadmium voltammetric signal occurred in the presence of a minimum 100-fold excess of the following ions: Al(III), Co(II), Cr(VI), Fe(III), Hg(II), Mg(II), Pb(II), and Zn(II). On the other hand, Cu(II) and Ni(II) caused a decrease in the cadmium signal when their concentrations were 20 and 25 times higher than the Cd(II) concentration, respectively.

### 3.5. Analytical Features

A calibration curve was obtained using the standard solution (0.1 mol L^−1^ acetate buffer, 1.5 × 10^−4^ mol L^−1^ Bi(III)) containing increasing concentrations of cadmium. The measurements were carried out using the following voltammetric parameters: activation potential of −2 V for 3 s, accumulation potential of −0.9 V for 50 s, and signal registration as a result of a change in potential from −1.0 to −0.4 V. A linear response was observed in the range of 2 × 10^−9^ to 2 × 10^−7^ mol L^−1^ Cd(II), as described by the equation y = 0.222x + 0.095, where y and x are the peak current (μA) and Cd(II) concentration (nmol L^−1^), respectively. The linear correlation coefficient was r = 0.998. The relative standard deviation (RSD) from five determinations of Cd(II) at a concentration of 5 × 10^−9^ mol L^−1^ was 3.5%. The detection limit estimated from three times the standard deviation for the lowest studied Cd(II) concentration and accumulation time of 50 s was about 6.2 ×10^−10^ mol L^−1^. Comparing these results with the performance obtained in other procedures described in the literature using electrodes modified with low-dimensional carbon nanomaterials (Table 1) clearly indicated that the presented work had the best sensitivity. As can be seen, carbon nanotubes were used in the vast majority of works, but only our work additionally introduced spherical glassy carbon, suggesting that it was this nanomaterial that contributed to achieving high sensitivity. Moreover, only our procedure provided an additional activation of the electrode introduced before the accumulation step as a result of applying a potential of −2 V for 3 s, which has also been shown to increase the sensitivity of the assemblies.

### 3.6. Application of the Elaborated Method

To examine the performance of the CNTs/SGC electrode modified with a bismuth film in a practical solution, the proposed method was performed to determine cadmium in certified reference materials: TM-25.5 Environmental Matrix Reference Material and SPS-WW1 Waste Water. To eliminate matrix effects, the standard addition method was used. The TM-25.5 Environmental Matrix Reference Material matrix contains up to 28 elements with their content ranging from 72 to 15.4 µg L^−1^, and only in the case of two elements is their content below 1 µg L^−1^. The concentrations of the 13 elements included in the SPS-WW1 Waste Water range from 20 to 2000 ng L^−1^, all of which are in excess in relation to cadmium, as follows: 100 times excess—Al; 50 times excess—Fe; Ni, P; 30 times excess—Zn; 20 times excess—Cu and Mn; 10 times excess—Cr; 5 times excess As, Pb, and V; and 3 times excess—Co. As can be seen, these materials represent the rich composition of environmental samples very well and their successful analysis confirmed the usefulness and validity of the proposed CNTs/SGC electrode (Table 2).

**Figure 6:**
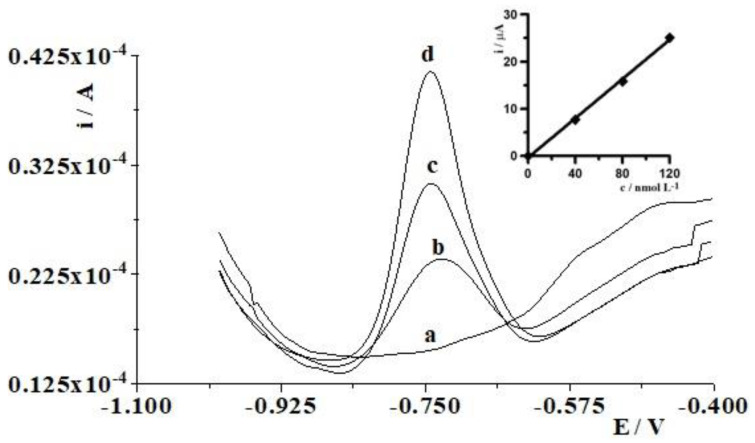
Differential pulse voltammograms obtained during Cd(II) determination in Bystrzyca River water samples: diluted five-fold (**a**); as (**a**) + 4 × 10^−8^ mol L^−1^ Cd(II) (**b**); as (**a**) + 8 × 10^−8^ mol L^−1^ Cd(II) (**c**); as (**a**) + 1.2 × 10^−7^ mol L^−1^ Cd(II) (**d**).

Using the optimal conditions, the proposed procedure was also examined for Cd(II) determination in water samples from the Bystrzyca and Bychawka Rivers spiked with Cd(II), as described in Section 2.5 Electrochemical analysis procedure. Figure 6 illustrates the exemplary voltammograms recorded in the course of cadmium detection in Bystrzyca River water samples. Three replicate determinations, using the standard addition method for each sample, were performed and the results are shown in Table 3. The recovery values ranged from 96.0% to 105.5% for Cd(II) with RSD ranging from 5.3% to 6.2%, attesting to the good accuracy of the proposed method and clearly demonstrating the applicability of this procedure for the determination of Cd(II) in a variety of river water samples.

## 4. Conclusions

This project focused on the fabrication of a CNTs/SGC electrode for use as an electrochemical sensor for metal ions in aqueous solution. A novel electrochemical sensor was successfully produced based on low-dimensional structures, spherical glassy carbon microparticles, and multiwall carbon nanotubes. To demonstrate the morphology of the CNTs/SGC electrode, an inverted metallurgical microscope and a high-resolution scanning electron microscope were used and the obtained images clearly showed spherical glassy carbon microparticles. The novel CNTs/SGC electrode modified with a bismuth film was assessed for the detection of Cd(II) ions in aqueous solution via the anodic stripping voltammetry technique. Under optimized conditions, the sensor achieved a limit of detection for cadmium at the μmol L^−1^ level and satisfactory precision. The detection of Cd(II) in certified reference materials (TM-25.5 Environmental Matrix Reference Material and SPS-WW1 Waste Water) and in river water samples also demonstrated that the presented sensor can be used for analyses of real samples with high accuracy and reliability.

## Figures and Tables

**Table 1:** An overview of electrodes based on low-dimensional carbon nanostructures used for the determination of Cd(II) by the ASV method. The publications are ranked according to decreasing limit of detection.

Modyfication Electrode Materials	Detection Limit (nmol L^−1^)	Accumulation Potential/Accumulation Time	Linearity Range (nmol L^−1^)	Ref.
carbon nanotubes/silver nanoparticles/bismuth nanomarticles	220	-/60 s	176–880	[46]
carbon nanotubes/Fe_3_O_4/_eggshell	21	−0.9 V/500 s	27–2222	[47]
carbon nanotubes/carbon natural halloysite	10.6	−1.2 V/90 s	100–10,000	[48]
carbon nanotubes/bismuth nanoparticles	9.4	−0.86 V/105 s	44–8889	[49]
carbon black	8	−1.1 V/300 s	6–1000	[50]
carbon nanotubes	6	−1.2 V/300 s	250–10,000	[51]
carbon nanotube/hydroxyapatite nanocomposite	4	−1.2 V/300 s	20–3000	[52]
carbon nanotubes	4	−1.1 V/300 s	40–4000	[53]
carbon nanotubes/bismuth oxide	1.96	−1.2 V/120 s	13–178	[54]
carbon nanotubes/spherical glassy carbon powder	0.62	−2 V/3 s−0.9 V/50 s	2–200	this work

**Table 2:** Analysis of certified reference materials TM-25.5 Environmental Matrix Reference Material and SPS-WW1 Waste Water. The samples were examined using the standard addition method.

Name	Material Type	Certified Cadmium Content (ng mL^−1^)	Cadmium Content Found (ng mL^−1^)	Recovery (%)	RSD (*n* = 3) (%)
TM-25.5	environmental matrix—solution	24.0	22.1	92.1	6.1
SPS-WW1	waste water	20.0	19.2	96.0	5.7

**Table 3:** Analysis of water samples from the Bystrzyca and Bychawka Rivers. The samples were examined using the standard addition method.

Sample	Cadmium Added (nmol L^−1^)	Cadmium Found (ng mL^−1^)	Recovery (%)	RSD (*n* = 3) (%)
Bystrzyca River	20.0	21.1	105.5	6.2
	40.0	41.8	104.5	5.5
Bychawka River	20.0	19.2	96.0	5.7
	40.0	40.8	102.0	5.3

## Data Availability

Not applicable.
